# Supplementary material for: Bacterial warfare is associated with virulence and antimicrobial resistance
Source: Nat Commun. 2025 Oct 22;16:9329. doi: 10.1038/s41467-025-64363-5 (PMC12546727; doi:10.1038/s41467-025-64363-5)
Supplement: Supplementary file 1 — Supplementary Information [file 41467_2025_64363_MOESM1_ESM.pdf]

Bacterial warfare is associated with virulence and antimicrobial resistance

Connor Sharp[1,2,3] and Kevin Foster[1,2,4]

1. Department of Biology, University of Oxford, UK
2. Department of Biochemistry, University of Oxford, UK
3. School of Biological Sciences, University of Reading, Reading, UK
4. Sir William Dunn School of Pathology, University of Oxford, UK

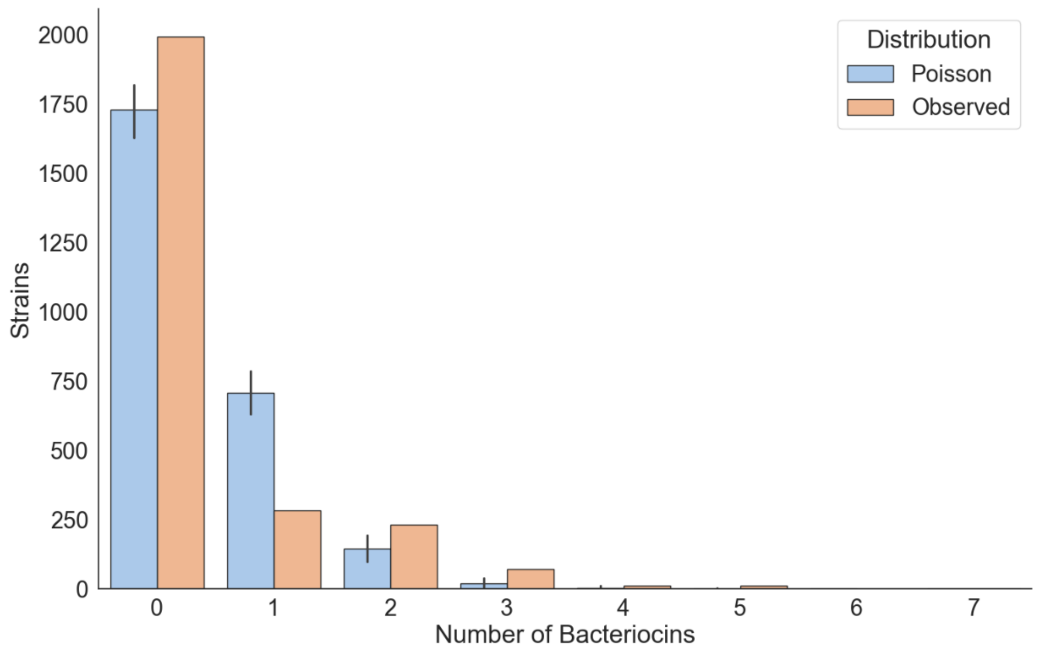

Supplementary Figure 1. Strains encoding multiple bacteriocins are observed more frequently than predicted by chance. The observed and theoretical Poisson distribution of bacteriocin distribution amongst strains in the dataset of 2601 *E. coli* genomes. Poisson distribution was calculated using the observed mean number of bacteriocins as λ. The Poisson distribution was approximated for a sample size of 2601 with error bars showing the min and max of 10,000 replicate approximations.

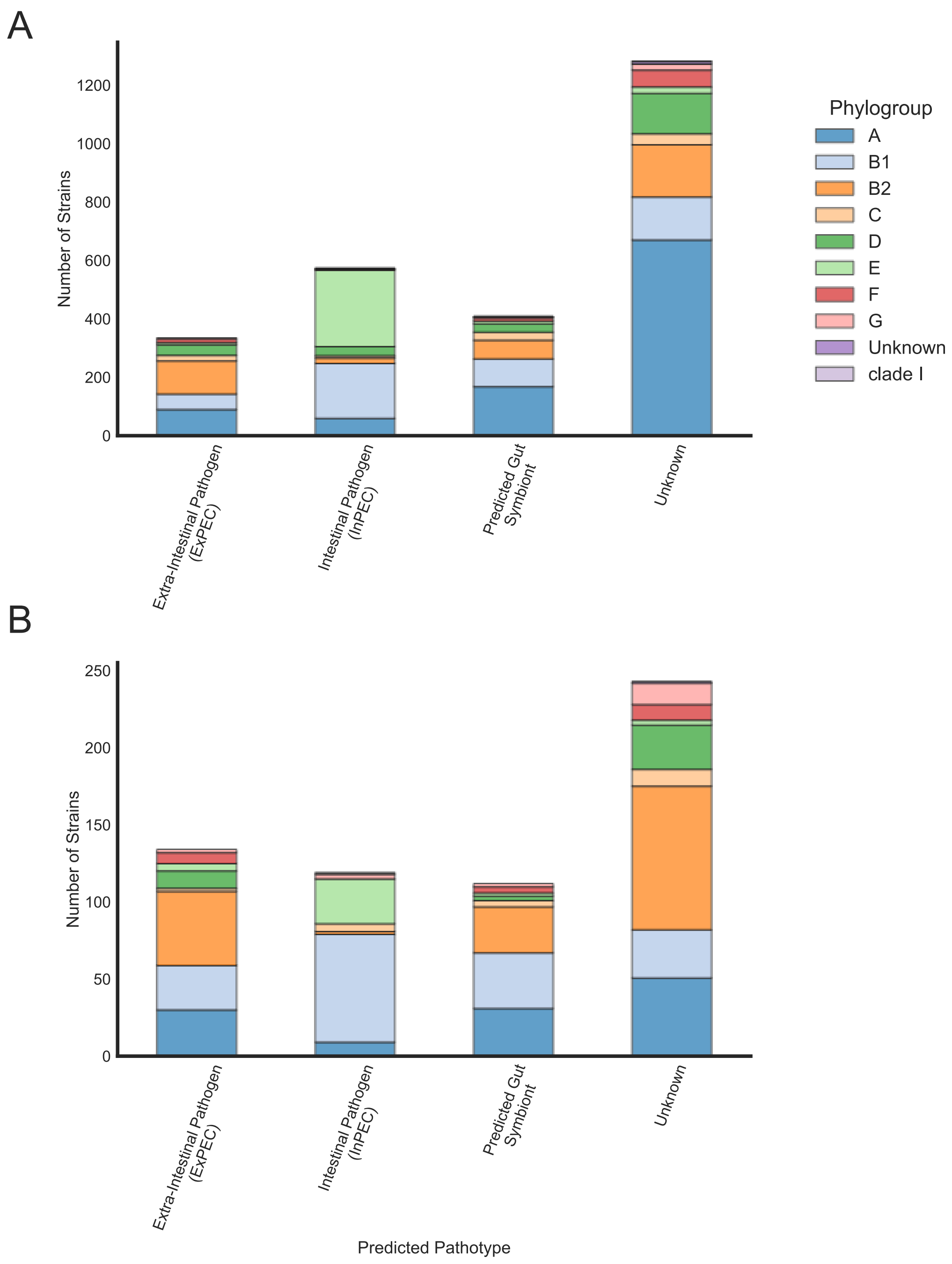

Supplementary Figure 2. Phylogroups are enriched in different pathotypes, including ExPEC strains which are mainly within the B2 phylogroup. A) Phylogroup and predicted pathotype distribution of 2601 *E. coli* strains. B) Distribution of phylogroup and pathotype in bacteriocin carrying *E. coli* strains.

A

B

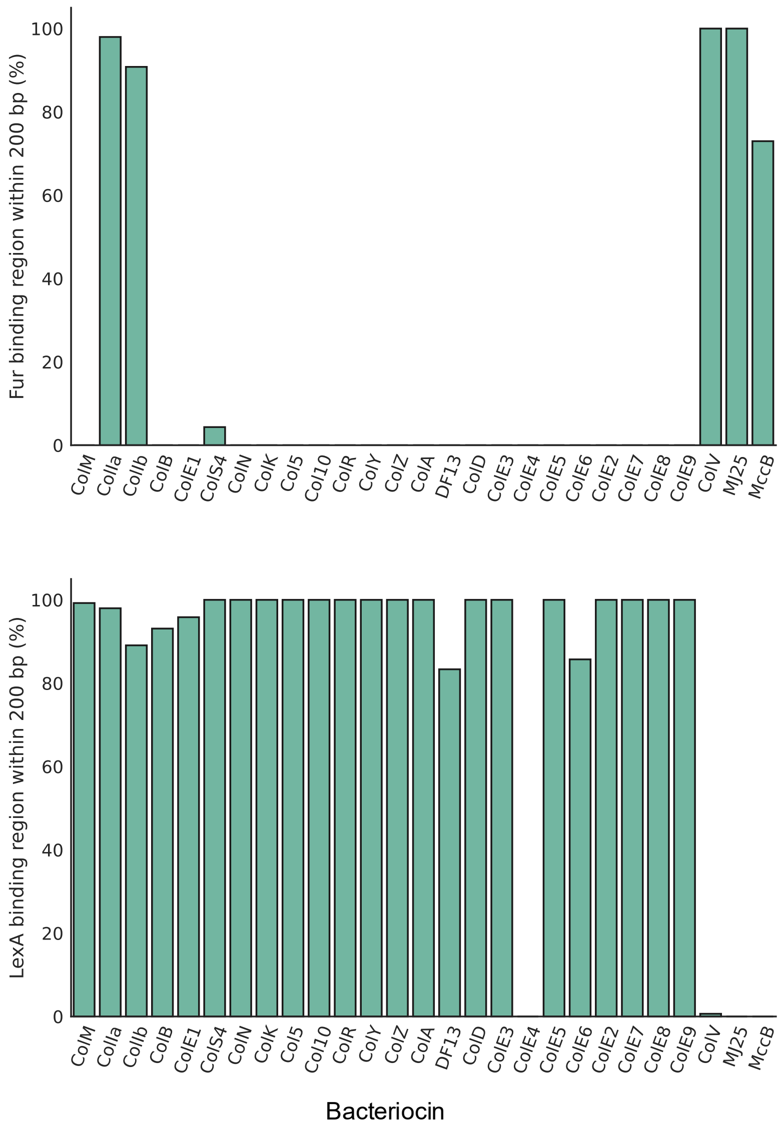

Supplementary Figure 3. Bacteriocins are regulated by combinations of LexA and Fur. A) Percentage of bacteriocin genes with an identifiable Fur binding region within 200bp of the predicted start site. B) Percentage of bacteriocin genes with an identifiable LexA binding region within 200bp of the predicted start site.

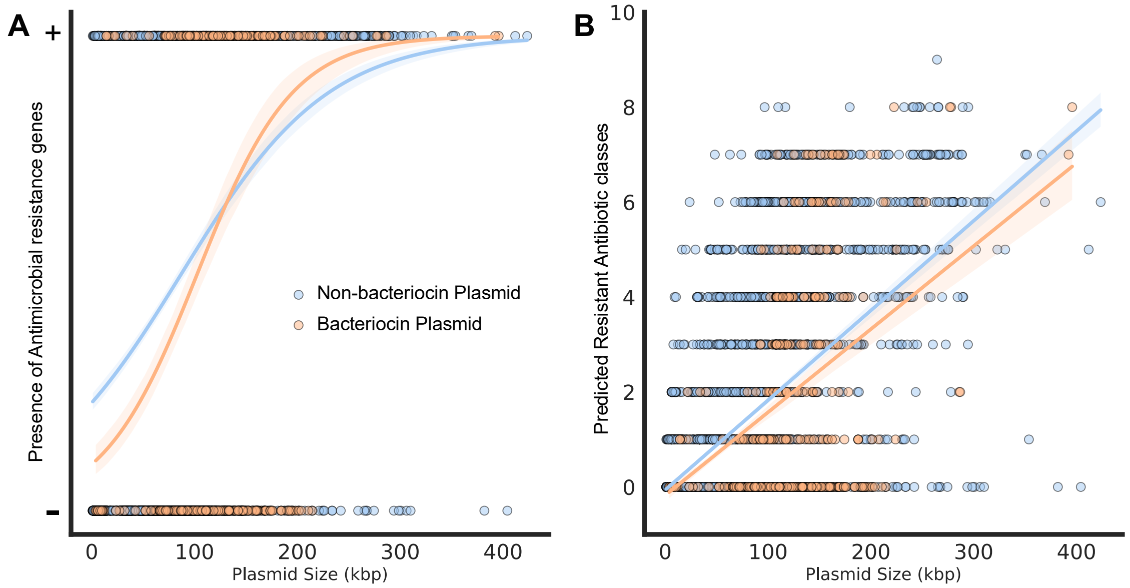

Supplementary Figure 4. Bacteriocins plasmids are not predicted to be enriched in antimicrobial resistance (AMR) genes compared to the E. coli plasmidome when corrected for their large size. A) Logistic regression model of AMR gene presence/absence of plasmids across the *E. coli* plasmidome. B) Linear regression model of the number of antibiotic class resistances provided by AMR genes in each plasmid as predicted by ResFinder.

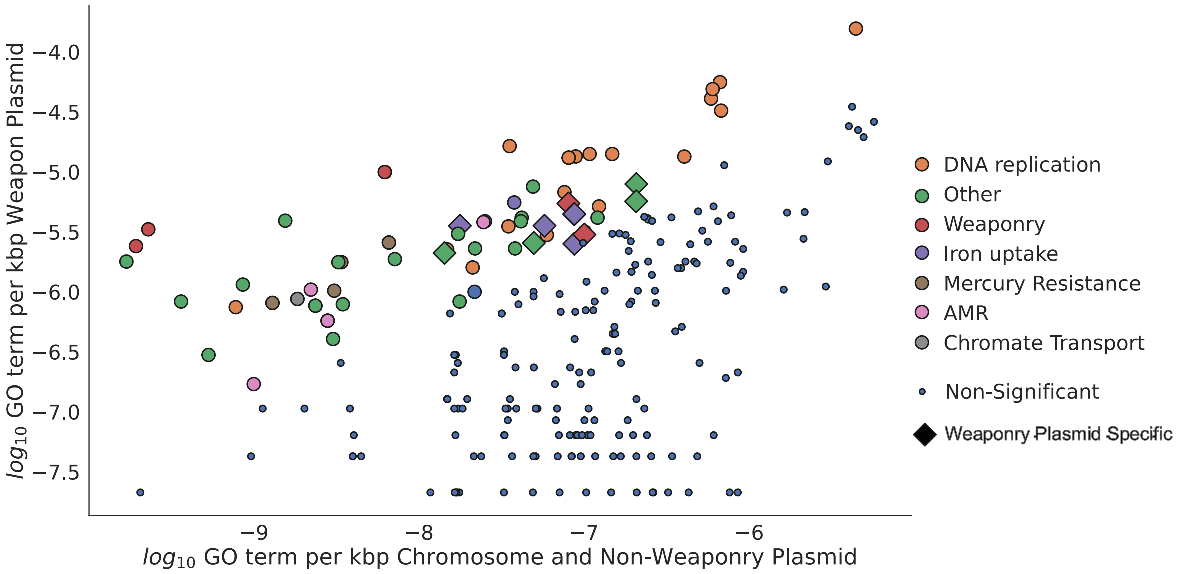

Supplementary Figure 5. Bacteriocin plasmids bring functions involved in bacterial competition, iron uptake and AMR into a cell. Analysis of the GO functions of genes in *E. coli*, both on plasmids and chromosomes, demonstrates that bacteriocin plasmids encode functions involved in iron-uptake, competition and AMR.

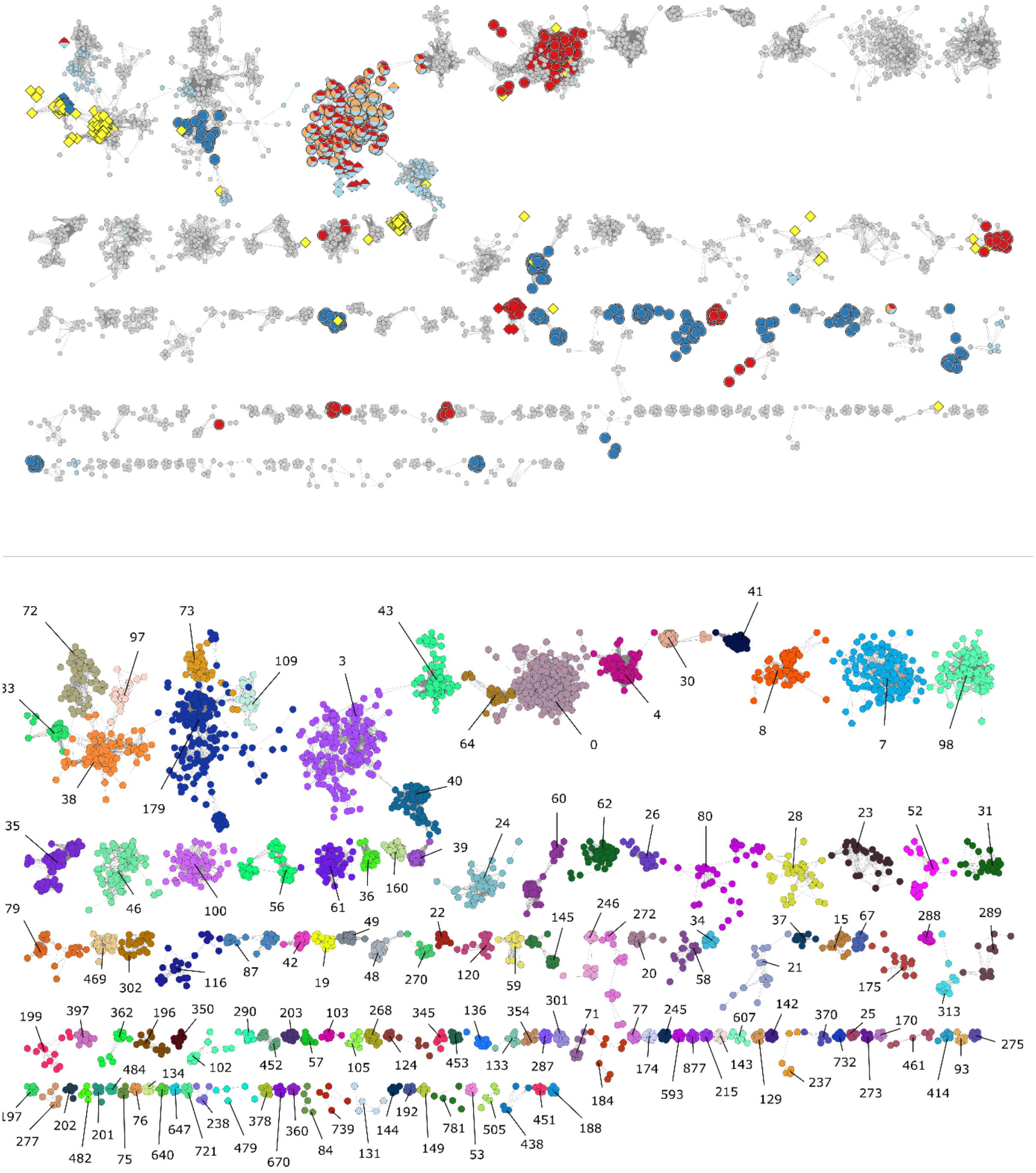

Supplementary Figure 6. Clustering of 5373 E. coli plasmids from PLSDB. Each circle represents an individual plasmid, edges indicate a Mash distance below the cut off (0.025). Only plasmid communities which contain [illegible]5 members are represented. Top: Bacteriocin clusters with genes of interest as shown in Figure 3D. Below: Plasmids clusters shown by colour with cluster number.

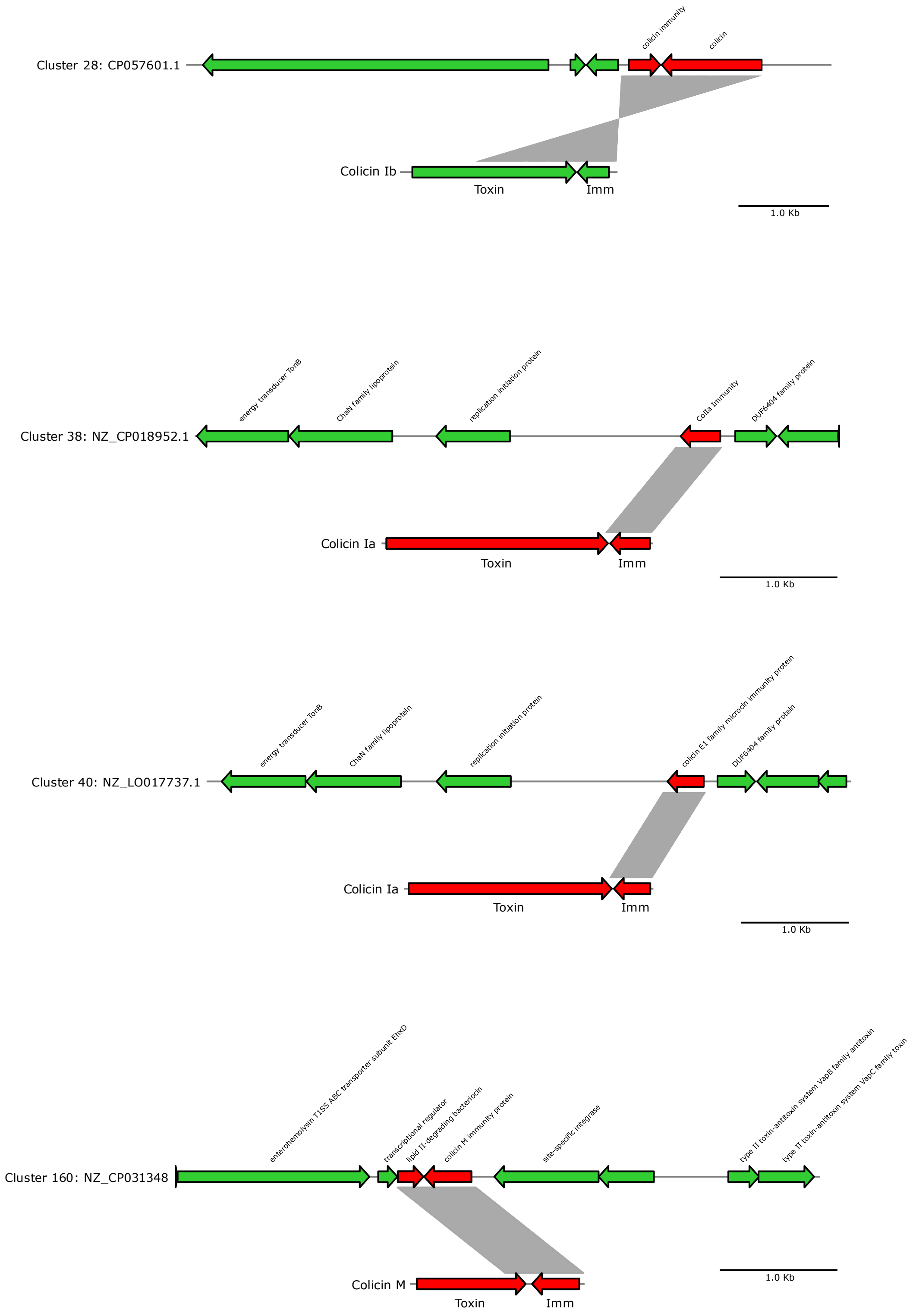

Supplementary Figure 7. Orphan immunity genes are encoded on multiple plasmids with either no colicin toxin gene or heavily degraded colicin toxin genes. Alignments of plasmids containing orphan immunity genes with the toxin-immunity

colicin pairs. Grey regions indicate alignments as identified by BLASTn with a threshold of 10% identity and 1000bp length.

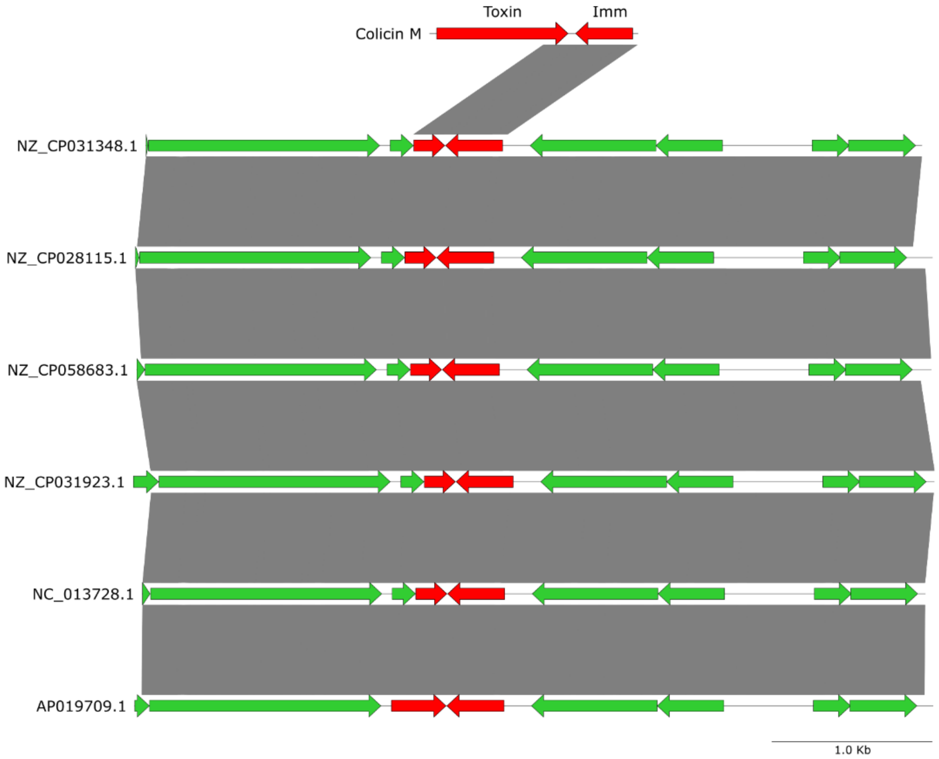

Supplementary Figure 8. Identical colicin orphan immunity genes and truncated colicins are identified in multiple distinct plasmids within a cluster. Alignment of the orphan immunity containing regions across multiple plasmids within the same cluster highlights that the immunity gene and truncated colicin is maintained. Plasmids were all different sizes and isolated from different environments (NZ_CP031348.1: Size - 89518 bp, Country of origin – USA, Niche – Cattle feces; NZ_CP028115.1: Size – 90123 bp, Country of origin – USA, Niche – Creek; NZ_CP058683.1: Size – 92578 bp, Country of origin - Germany, Niche – Salad; NZ_CP031923.1: Size – 95298 bp, Country of origin – Canada, Niche – Human feces; NC_013728.1: Size – 111481 bp, Country of origin – Australia, Niche – unknown; AP019709.1: Size 86874 bp, Country and origin – Japan, Niche – Human feces.)

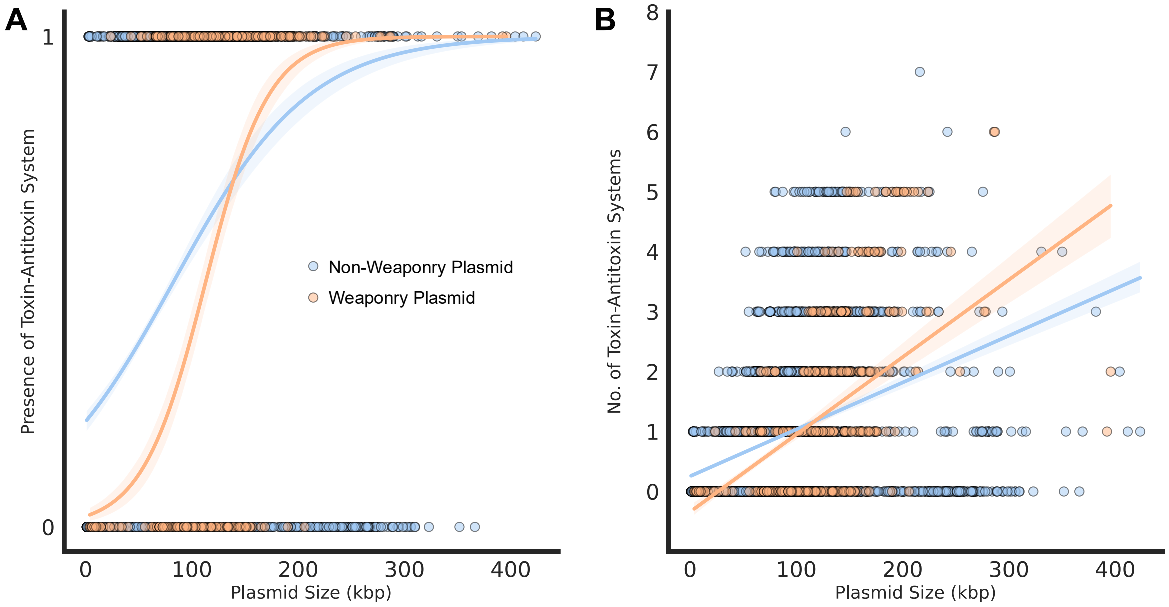

Supplementary Figure 8. Bacteriocin plasmids are not less likely to encode a Toxin-antitoxin system or encode fewer Toxin-antitoxin systems. A) Logistic regression model of the presence of Toxin-antitoxin systems on plasmids in the *E. coli* plasmidome. B) Linear regression showing the number of predicted Toxin-antitoxin systems on plasmids within the *E. coli* plasmidome accounting for plasmid size.

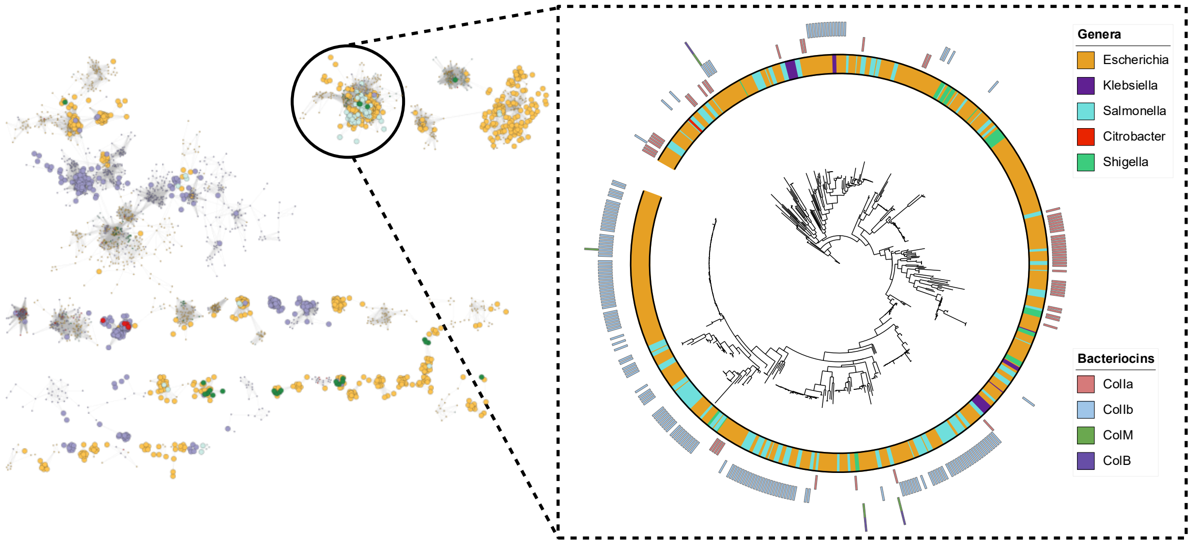

Supplementary Figure 10. Plasmid clustering reveals similar plasmids shared between *Salmonella*, *E. coli* and *K. pneumoniae*, suggesting that plasmid host range does not limit the sharing of bacteriocin plasmids between the three species. Analysis of a plasmid cluster which contains bacteriocin and non-bacteriocins plasmids shared between all three species. Phylogeny was calculated from conserved genes on plasmid backbones.

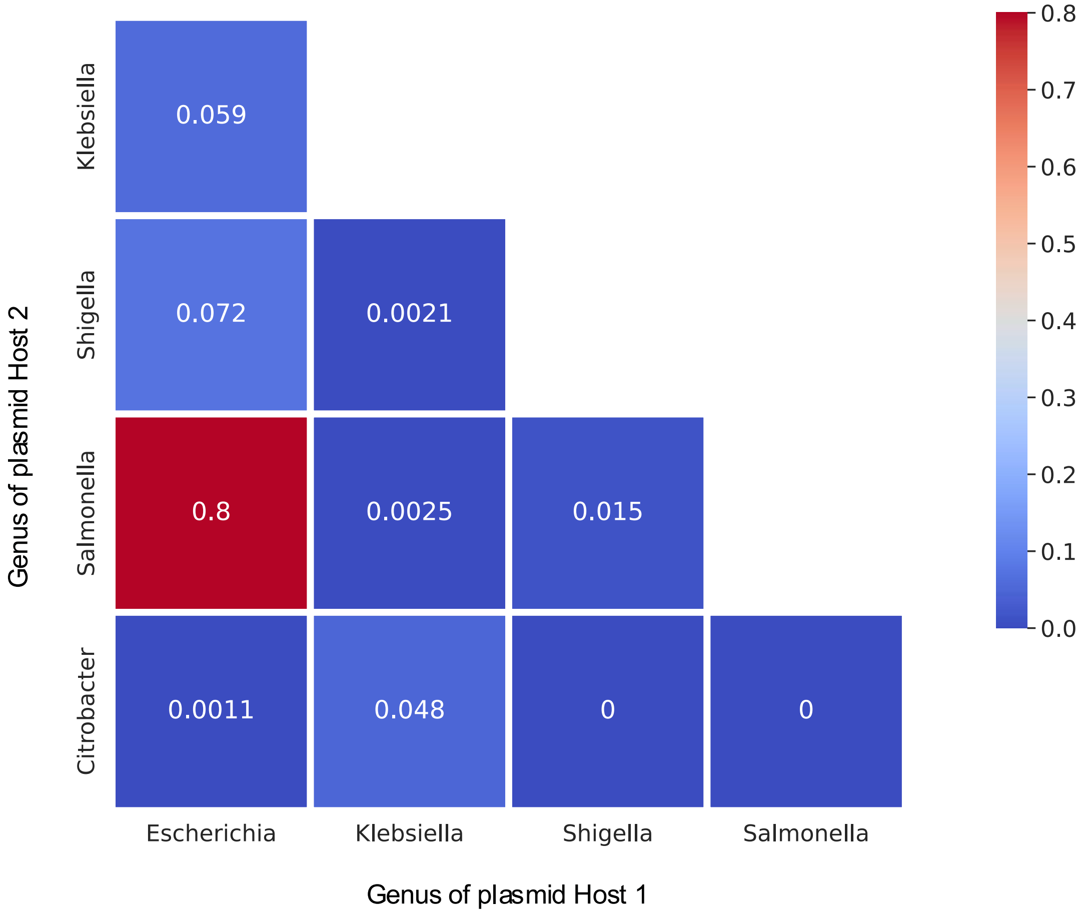

Supplementary Figure 11. Heatmap of edges (indicating related plasmids) between different genera in figure 5. The majority of edges between genera are between *Escherichia* and *Salmonella* plasmids.

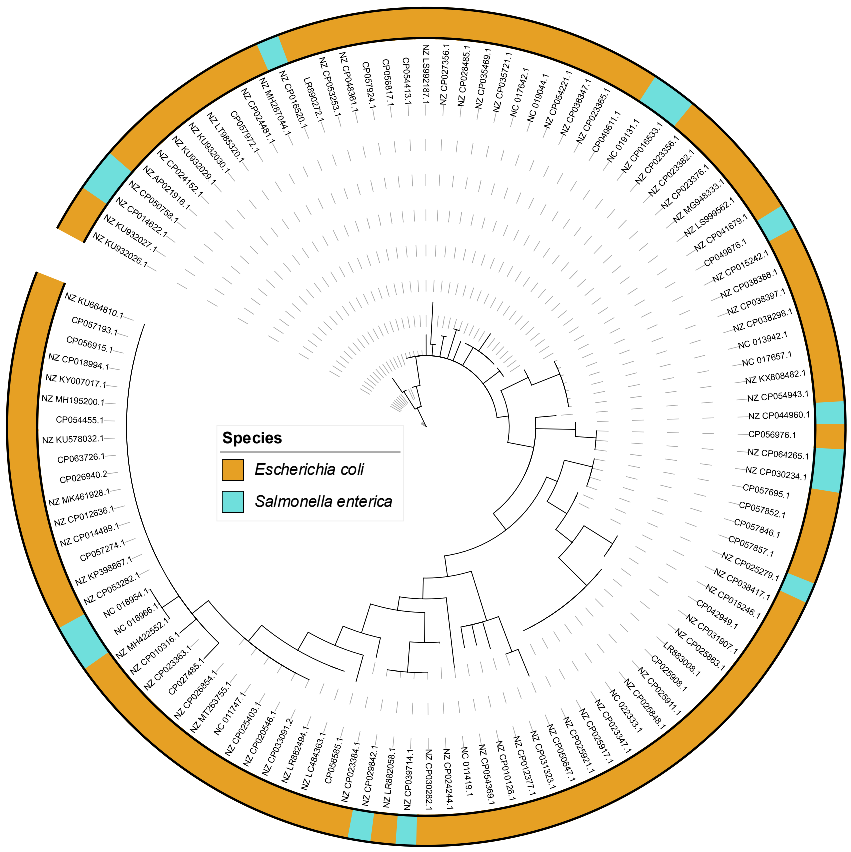

Supplementary Figure 12. Phylogeny of Colicin Ia suggests genes have been shared multiple times between *Salmonella* and *E. coli*. Phylogeny of concatenated Colicin Ia and immunity genes from *E. coli* and *Salmonella*.

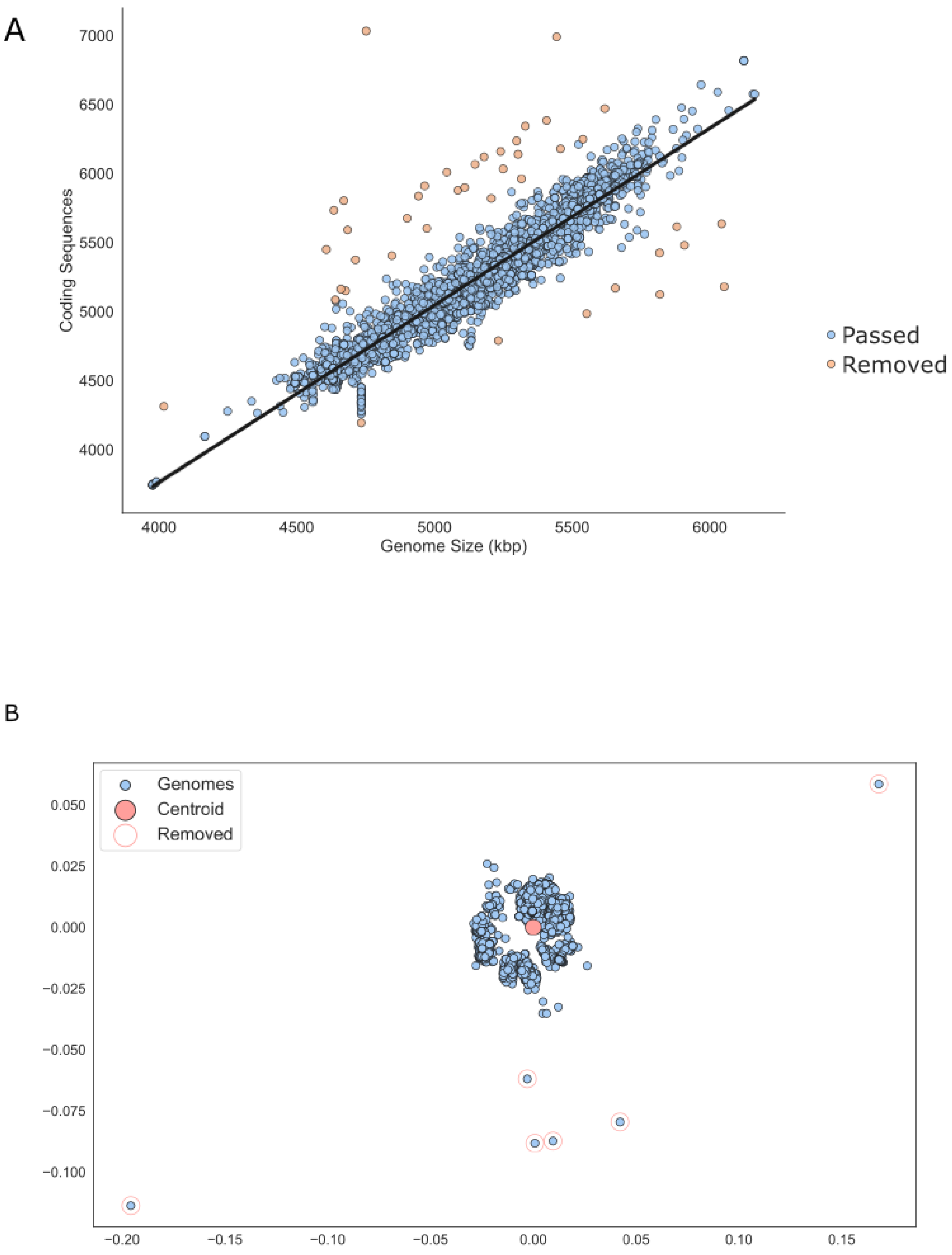

Supplementary Figure 13. Pre-processing of E. coli pangenome strains to remove contamination. A) E. coli genomes which deviated from a linear relationship between genome length and number of coding sequences were removed from further analysis. B) Possible contaminated genomes were removed by analysis of MASH distances between all strains in the population. After MDS scaling, outlier strains were removed.

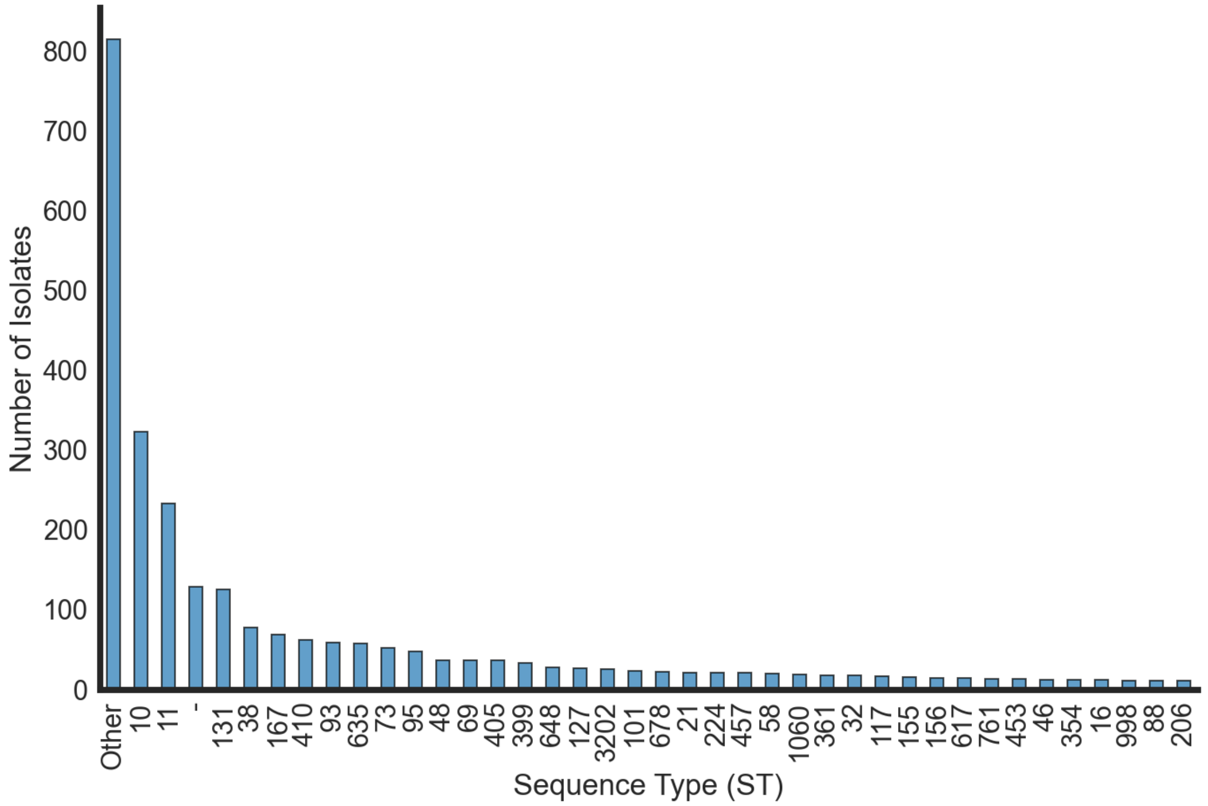

Supplementary Figure 14. Distribution of sequence types in our dataset of 2601 *E. coli* genomes. Our dataset includes 438 different sequence types, with 38 STs containing more than 10 strains. STs with 10 or fewer strains are represented by ‘Other’ (400 STs, 814 strains) . ‘-‘ includes 128 strains which could not be sequence typed.

Supplementary Table 1. Sequences used to identify bacteriocins in *E. coli*.

Bacteriocin Gene	Accession
ColM	WP_000449473.1
ColM immunity	WP_105457450.1
ColIa	WP_001582575.1
ColIa Immunity	WP_001080729.1
ColIb	WP_010891256.1
ColIb Immunity	WP_000762570.1
ColB	WP_021539362.1
ColB Immunity	WP_032084022.1
ColE1	P02978.1
ColE1 Immunity	WP_000058760.1
ColS4	CAB46008.1
ColS4 Immunity	WP_104807860.1
ColK	WP_159420795.1
ColK Immunity	WP_011264161.1
Col5	WP_000362089.1
Col5 Immunity	WP_011264161.1
Col10	Q47125.1
Col10 Immunity	MCD4231352.1
ColR	WP_104355620.1
ColR Immunity	WP_040090309.1
ColY	WP_010892543.1
ColY Immunity	WP_021534853.1
ColZ	WP_172694321.1
ColA	WP_008323639.1
ColA Immunity	EEW1531982.1
CloDF13	WP_010891190.1
CloDF13 Immunity	RRE37366.1
ColD	WP_016245160.1
ColD Immunity	WP_001038407.1
ColE3	WP_000012964.1
ColE3 Immunity	WP_000523346.1
ColE4	CAA45167.1
ColE4 Immunity	RRE37366.1
ColE5	WP_015420185.1
ColE5 Immunity	ELC99665.1
ColE6	WP_052980126.1
ColE6 Immunity	WP_001419702.1
ColE2	WP_172690082.1
ColE2 Immunity	WP_000421100.1
ColE7	WP_021530049.1
ColE7 Immunity	WP_000420692.1
ColE8	WP_012766032.1
ColE8 Immunity	WP_000421100.1
ColE9	WP_012644886.1

ColE9 Immunity	WP_012644887.1
mchA	AJ009631
mchS1	AJ009631
mchS2	AJ009631
mchS3	AJ009631
mchS4	AJ009631
mchX	AJ009631
mchI	AJ009631
mchB	AJ009631
mchC	AJ009631
mchD	AJ009631
mchE	AJ009631
mchF	AJ009631
mchI	AJ009631
mcmI	AJ515252
mcmA	AJ515253
mcmM	AJ515254
mcbA	FM877811
mcbB	FM877811
mcbC	FM877811
mcbD	FM877811
mcbE	FM877811
mcbF	FM877811
mcbG	FM877811
mcjA	AF061787
mcjB	AF061787
mcjC	AF061787
mcjD	AF061787
cvaC	AF062848
cvaI	CP005931
cvaA	CP005931
cvAB	CP005931

Supplementary Table 2. Additional sequences used to identify Colicin-like proteins in *Escherichia, Klebsiella, Salmonella, Citrobacter* and *Shigella*.

Colicin-Like protein	Genus/Species of origin	Accession
SalE2	*Salmonella*	KTM78572.1
SalE3	*Salmonella*	GAS18013.1
SalE7	*Salmonella*	KSU39545.1
SalIa	*Salmonella*	OIN35410.1
SalIb	*Salmonella*	OIN32443.1
ColA-like protein_KP	*K. pneumoniae*	SAV78255.1
Colicin-like protein	*K. aerogenes*	WP_063414841.1
Colicin-like protein	*K. oxytoca*	WP_024273778.1
Colicin-like protein	*K. variicola*	KDL88409.1
Colicin-like protein	*K. pneumoniae*	BAS34675.1
Colicin-like protein	*K. pneumoniae*	EWD35590.1
ColM-like protein	*Klebsiella*	WP_047066220.1
ColM-like protein	*K. variicola*	CTQ17225.1
ColM-like-protein	*K. aerogenes*	WP_015367360.1
Klebicin B	*K. pneumoniae*	AGT26949.1
Klebicin C	*K. penumoniae*	AAT85004.1
Klebicin D	*K. oxytoca*	WP_224261359.1
