# Supplementary material for: Bacterial warfare is associated with virulence and antimicrobial resistance
Source: Nat Commun. 2025 Oct 22;16:9329. doi: 10.1038/s41467-025-64363-5 (PMC12546727; doi:10.1038/s41467-025-64363-5)
Supplement: Supplementary file 2 — Description of Additional Supplementary Files [file 41467_2025_64363_MOESM2_ESM.pdf]

Title: Supplementary Data 1.

Description: Excel compatible CSV file containing annotations of *E. coli* genomes associated with Figure 1.

Title: Supplementary Data 2

Description: Excel compatible CSV file containing annotations of *E. coli* plasmids from the PLSDB associated with Figures 2-4.

Title: Supplementary Data 3

Description: Excel compatible CSV file containing annotations of plasmids from *Escherichia, Salmonella, Klebsiella, Citrobacter* and *Shigella* species associated with Figure 5
